# Why the Profession Should Adopt Porcelain as a Standard Filling Material

**Published:** 1907-04-15

**Authors:** W. T. Reeves

**Affiliations:** Chicago, Ills.


### WHY THE PROFESSION SHOULD ADOPT PORCELAIN AS A STANDARD FILLING MATERIAL.

BY W. T. REEVES., D.D.S., CHICAGO, ILLS.

Read before the Michigan State Dental Association, at Detroit, July 10, 1906.

*Definition*, that which has accuracy or authority; serving as a gauge, test, guide or model; hence, a very high or excellent kind of type. Any type, model, example, or authority with which comparison may be made; any fact, thing or circumstance forming a basis for adjustment and regulation; in fact, a criterion of excellence.

Having found out what is required of a standard we will see if porcelain fills those requirements better than any other material in our dental armamentarium.

Anything to become a standard must possess the greatest number of points of excellence whereby it becomes the gauge, test, guide, model, example or authority with which comparison may be made, *a criterion of excellence.*

Dentistry as a profession is varied and far reaching, but that branch of dentistry designated as Operative Dentistry has for its sole end or objective point to be obtained, the restoration to as near a normal condition as possible (by the use of foreign materials) the loss of tooth substance caused by caries or other accidents. It is but fair in making a choice of what shall be the material to be the standard that we take average conditions. We should not be hampered in the choice of material by time, finances or prejudices, but be given carte blanche to use what will result in the greatest good for all time, or in other words, to use that material which will make the filling par excellence in the largest numbes of cases.

Gold for a great many years has occupied the position of being the standard by which all materials were judged as tooth savers ,but how short it comes of being ideal you all know. Whith the advent of porcelain and the modern technique that has been developed in the past fifteen years, we have a material that equals most of the good points of gold, surpasses many and has some that gold does not possess. I will try to enumerate the good points of gold and porcelain and compare the two materials as we go along. First of all, a material to be a standard must possess the quality of permanency to a high degree and to be permanent it, first of all, must seal hermetically the cavity so that the fluids of the mouth cannot seep in and permit recurrent decay. How sadly gold fails in this respect you all know by the many leaking gold fillings that come under your observation every year of your practice and from the hands of the best operators. There are gold operators who claim that they can pack gold into a cavity and seal it hermetically, but a simple experiment will prove the fallacy of this. I do not believe there is a gold enthusiast who will claim that he can pack gold into a cavity and make a joint between gold and cavity walls as close as the joint of fused porcelain upon platinum. If you are stripping the platinum matrix from an inlay and it is coming off hard, if you will dip it into water the water will penetrate between the platinum and the porcelain and it will then come off easily. With the use of porcelain to fill the cavity the joint is completed with cement and as long as the cement remains the cavity is hermetically sealed. What that means you can judge.

The average life of a gold filling is placed at flve years. Now there must be a great many gold fillings lasting a great deal longer to bring up the average to five years. You hear about gold fillings that have been in twenty, thirty, forty or fifty years. The forty and fifty year old fillings are rare and it is possible that anything of a fair quality of workmanship would have stood just as well, as it is quite likely that the mouth was immune to caries. We will consider the fillings that have stood twenty and thirty years and are or were apparently as good as the day they were put in. With them when the end came the tooth like the "one horse shay" simply crumbled all to pieces leaving nothing but crowning possible, and not always that. With porcelain no such condition is possible as long as the filling remains seated. The cement joint hermetically seals the cavity and there can be absolutely no decay under the filling. The worst that can happen with a porcelain filling is its breaking loose from its attachment. When that occurs the patient knows it; the trouble can be quickly remedied by resetting the filling and no harm results. But how about the gold fillings that do not last five years ? How many of such you see every year and what a sad commentary upon our skill as a profession trying to save teeth! Take the conditions as we find them every day in our practice, and it is safe to say the majority of our patients are abnormally susceptible to caries. Take the most desperate of these cases where the concientious dentist hesitates as to what to do, if porcelain is used it will be found to be practically a permanent filling. It is from this desperate class of cases that I have started in to preserve by using porcelain. I have made fillings which have stood for over five years and up to ten and having stood that long I can see no reason why they should not continue for as many or more years. From them I draw my conclusions as to permanency of porcelain as a filling material, for if it will do this under the most unfavorable conditions, it will do as well, or better, under more favorable conditions. I believe, therefore, we can score one for porcelain.

For restoring contour, contact points and the interproximate space, gold is good, but porcelain is better. There is no doubt in the world but that better tooth contour and form can be restored with porcelain than with gold, and for contact point ,porcelain is simply ideal . For preserving or recovering the interproximate space, I believe more is done and can be accomplished with porcelain than was ever attempted with gold; for one of the first requisites to using porcelain is working space. You cannot put in flat porcelain fillings and you ought not to put in flat gold fillings. Working space should be obtained and lost space regained by wedging, previous to attempting any operation. Having such space there is no temptation to stop short of full contour and perfect contact point. With porcelain you can build out to full contour just as well as to stop short of it, but with gold it may mean an additional hour or more of hard labor which neither you nor your patient is able to stand.

Porcelain has brought benefit to the patient in more ways than one, as well as to the operator. Working space being essential to success separation is practiced. The operator noting the benefits that have come to the patient by proper contouring of his porcelain work and working to this ideal, has his moral stamina raised and continues to separate when other materials are to be used. For restoring tooth form with absolutely smooth contact points and for preserving,and protecting the interproximate space, porcelain is unquestionably superior to gold.

Edge strength is a quality that has been loudly talked about and credited to gold only, because all other materials carried to attenuated edges crumble and brake. Porcelain fillings having attenuated edges are very friable and cavity formation that would necessitate attenuated edges cannot be practiced.

Edge strength sounds good and theoretically is good, but practically it is a menance to the filling that depends upon the edge strength of the gold and not on the cavity formation. Why is the modern and scientific cavity preparation for gold such that it does not call upon the edge strength of gold for one of the requisite qualities? Because the fillings having attenuated edges of gold, while they will not break or crumble, under masticatory or other force will curl up on leaving the tooth ever so slightly and results in a leaking filling in the early stages of its existence.

Porcelain cavity preparation should be such that there are no attenuated edges, but above and beyond all this porcelain must be properly fused to secure its greatest strength. Today in all lines of porcelain work more harm results and more failures come through the overfusing of porcelain than from all other causes combined, but if the material is abused in baking failure should not be charged to it, but to the inexperience of the operator.

When the color is considered there is no comparison as to the qualities of the two materials. Porcelain can be made to duplicate all the varied shades of the teeth so that at conversational range a restoration cannot be detected. Gold is a contrasting color and its glitter can be seen as far, and often farther than other features about the face can be distinguished. That gold is tolerated at all in the mouth is one of the strange things when you come to think of it. It is through indifference largely that gold is tolerated. The American people have become accustomed to seeing gold in the mouth of the majority of their fellows and constant association brings indifference and tolerance. Some will say that patients like the looks of gold, that they want it, because it makes a showing for the money they have spent on their teeth. Contrasting colors would not be tolerated in the restoration of any other lost organ. Suppose a pronounced blonde with blue eyes lost one of them and a glass substitute had to be inserted and that person being a great admirer of black eyes should insist that the glass substitute be a black eye. Such a restoration would look like———; yes, that is what it would look like, and restoring lost tooth substance with gold looks about as badly. Refined people are fast finding out that something can be used instead of gold and that it will preserve the teeth and they are demanding that kind of filling. This is what is going to bring porcelain into general use. These are the main points upon which gold has depended to maintain the position of being considered the standard filling. In addition to these, porcelain possesses several points of excellence that gold does not possess at all. I will briefly mention two or three of them.

Porcelain possesses to a very high degree the quality of compatibility to live tooth. Gold does not possess that quality at all.

All materials used for filling teeth are supposed to protect the teeth from caries, but porcelain is the only material that brings immunity to adjacent tooth surfaces. The area of tooth surface that is replaced by glazed porcelain brings an increased percentage of immunity to adjoining tooth surfaces; glazed porcelain will not collect or retain bacterial plaques or gelatinous deposits. All other surfaces, whether tooth, gold or what not, will retain these plaques and deposits and consequently endanger adjoining tooth surfaces. Approximating teeth having one or more of the surfaces replaced with glazed porcelain are protected from caries fully 50 per cent. In other words, the glazed porcelain brings 50 per cent more immunity to an approximating tooth surface.

The most important consideration of all is the material aid porcelain brings in the work of preventive dentistry, the so-called *prophylaxis.* Where any lost tooth substance is to be replaced porcelain is the ideal material with which to make such restoration, for it will do more than any other material in bringing about the ideal prophylaxis conditions so much desired. Those who are becoming porcelain workers and have entered through the prophylaxis doorway are likely to become the most enthusiastic porcelain men of all.

I shall now leave it for each of you to decide what you will choose to be your standard filling material. As for me and my kin we will hoist the banner of porcelain and follow that standard on to a glorious victory over caries.

I want to digress from the title of the paper slightly, and leaving out the word standard tell you why the profession should adopt porcelain for filling teeth.

It should do so to keep up with the times. Do you know of anyone who has mastered the techniqne and can use porcelain successfully, who is losing practice? On the other hand, do you not know that all these men are increasing their practices? At the recent Illinois State meeting there were three porcelain men, and three others who did not use porcelain, dining at the same table. The conversation turned to porcelain and the question was asked. "Would you advise me to take up porcelain filling?" The most conservative porcelain man in Chicago replied, "Porcelain has doubled my practice." That is a conservative estimate of what it has done for some of those who have gone into porcelain practice If it will do that for others, it will do as much for you.

Do not let the cement question frighten you. It is a "bug-a-boo" raised by those who are afraid to go into porcelain. It is true that nothing is stronger than its weakest point, and that cement is called the weak point of inlay work, but with its weak point it is much stronger than all that has gone before To-day I think no more about having to reset a porcelain filling than you do when you have to reset a crown or bridge. I will cite the most extreme case of resetting in my practice. Patient A, 22 porcelain fillings work begun June 1, 1899; 13 resettings, confined to 5 or 6 fillings. A year ago I told her that I would not blame her if she was discouraged with porcelain fillings and if she so desired we would put in gold. Her reply was, "I don't want anything but porcelain if I am where it is possible to have porcelain put in. It is not half the bother to me that it was to have my gold fillings decay and have to be refilled." On the other hand is patient B—and this is only an average case, for many patients never have a filling to be reset—43 porcelain fillings put in during January, February and March of 1901; three to reset and only three new cavities in that mouth since that time. How is that for prophylaxis.

You can do more work with porcelain in a given time than you can with gold. Taking the work as it comes in your practice day in and day out, large and small fillings, when progressed to the point that you do 50 per cent in porcelain and 50 per cent in gold you will have gained a technique that will enable you to do the porcelain fillings as rapidly as you can do the gold, and when you have passed beyond that per cent, up to 75 to 90 per cent of the work with porcelain, you will be able to save at least 25 per cent in time, or, to put it the other way, do about 33 1-3 per cent more work. At a very conservative estimate on the average you can get 25 per cent better fees for porcelain than you can for the same work in gold. Now, this is nothing but a simple matter of cold figures and if you will put them down and see what it means to you at the end of the year, I think you will decide that it is best for you to get into porcelain.

What would an increase of one-third in the output usually mean? Longer hours or harder work. But using porcelain and gaining this increased output, means just the opposite, for the demand on nervous energy and physical strength is so greatly lessened for both operator and patient that work becomes almost a pleasure, and when work ceases to be a drudgery you enter upon it with all your faculties at their best and you work to ideals with a fervor and zest that brings its own reward.

### DISCUSSION.

*Dr. C. H. Cudworth*, Wilwaukee, Wis.: In genaral I agree heartily with the ideas Dr. Reeves has expressed in his paper, but I think he exaggerates a little in his selection of the title for his paper. If he had said an inlay was the standard filling he would have come nearer to covering the ground.

Porcelain has its place, and, in my judgment, with rare exceptions, its place is confined to the six anterior teeth and to the buccal cavities of the rest of the teeth of the mouth. It has not been my experience that porcelain was the proper filling for cavities that have to resist a large amount of strain of mastication. I believe that in cavities of that kind the edges should be strong, which can be obtained only by the use of the gold inlay.

There is considerable difference in the preparation of a cavity for the gold inlay and for the porcelain, for we can bevel the margins of our cavity so as to obtain the greatest amount of strength possible to obtain with any material known by us ing a gold inlay, because it presents a greater resistance to the stress of mastication than the ordinary filling.

In the matter of the dropping out of the gold inlays, or inlays of any kind, I believe that we have improved our technique. I believe that cavity formation is the secret of permanency of the porcelain or gold inlay Of course, as Dr. Reeves has said, the cement problem is the one that we are "up against" just at present. e have cements that are giving us greater success with our inlays, and the manufacturers of cements are year by year increasing the durability of their product, and I think are trying hard to give us a cement that will be as permanent as the filling itself. When they have accomplished that, we shall arrive at the stage where we can adopt the inlay as the standard filling, but until that time comes we cannot be positive about it. Another feature of the cement problem that makes the inlay lack perfection is the refraction of light. When the inlay is set the infiltration of the cement with pigments from the saliva practically spoils the translucency of the inlay.

*Dr. Geo. F. Burke*, Detroit: I believe that it is very important in this work that one should start right, and the best way is to take the course from an experienced expert in order to get the best results. Those who take it up themselves work in the dark for a long time without understanding the underlying principles The separation of the teeth, preparation of the cavity, burnishing of the matrix and fusing of the porcelain, and finally setting of the inlay are procedures that we do not have to contend with in any other form of filling material. Now, if a person does not take a post-graduate course he must work along for a number of years in the dark, and I do not believe he ever acquires confidence or secures adequate results. In my experience I find that it takes about twice as much time to do good porcelain inlay work as it does to fill with gold, therefore, I charge twice as much for the porcelain as I do for the gold, and that is the reason probably why I do not do more of it. A great many of my patients cannot afford to pay for porcelain. I have not worked long enough in it to develop the speed which Dr. Reeves claims.

*Dr. J. M. Thompson*, Detroit: There is one place that I want to commend for the use of porcelain. If some of the dentists who are cutting down good teeth and slipping abominable gold or porcelain crowns over them, when they do not need to do so, would use gold or porcelain inlays their patients would appreciate them more, and it would be better all around.

As far as the cement question is concerned, I think that a little stress should be laid upon the foundation upon which we put porcelain or gold. If a tooth has reached that stage of decay where it approaches the pulp very closely, and we find it is necessary to remove a great deal of infected dentin, I do not believe in filling the tooth half full of cement and then putting in a porcelain filling on top of that. The pulp will die under the cement filling, in spite of all you can do. You will find that the figures Dr. Reeves gave you in regard to time being saved and practice being doubled practically correct. I can verify that from my own experience, for not only has my practice increased considerably, but, better still, my health has greatly improved.

*Dr. Douglas*: I can show you gold fillings, without leaving this room, that have been in from 52 to 53 years and are good now. They were made with soft gold. I met a lady this morning when I was coming down to the city. She said she had a filling in one of her front teeth that I put in forty years ago. I frequently meet such people who speak in commendation of gold filling, with soft gold. I doubt whether there are many in this room that can speak as well forty years from the time they put in cohesive gold fillings.

Dr. Howe, of the City of Mexico, showed me a tooth taken out of an old burying ground in the Argentine Republic. It had an inlay filling. The filling was of jet, a kind of stone found in China. It was cemented in with a white cement. If they could make a cement so many years ago that woul last as long as that lasted, don't be so afraid of cement that you cannot use cement in putting in porcelain or gold inlays.

*Dr. Land*: I have been annoyed, not because the cement or inlay came out, but because the patients complained of the dark line at the joint. If inlays are not as substantial as porcelain crowns and bridges then you ought to hesitate before using cement in any crown. All Logan, Davis and other pivot crowns always are better when put in with an intermediate stratum of cement. Because you do not hide the joint is no reason why decay would not occur around this filling any more than around any crown cemented on any bridge. ..If you should put on the rubber dam and hunt for the joint you would be surprised as to how much the cement has been washed out. I cannot abondon the gold or amalgam or any other kind of crown because of this. I have been criticised because I left the matrix on, but I want to tell you that those fillings have stayed, though showing the dark joint and the little line of platinum. Where most needed, and for technical results we must use block putties. These putties can be put through the furnace six or eight times without injury to colors. They can be carved, and are three times as strong as any of the bodies now used. A low fusing putty is not as strong as a high fusing putty. They must be fused in a stronger than white heat if we can get it. For ideal results we must have a furnace that will fuse a higher grade of porcelain than any that is now on the market. Every bit of artificial flux reduces the fusing point and weakens the porcelains and makes them opaque and takes away their translucency.

*Dr. Reeves, closing discussion:* In regard to what Drs. Cudworth and Thompson said,that if I had said inlays instead of porcelain, I might say that your program trustee selected the subject for me to write upon. A material to be a standard by which to compare other materials for filling teeth must possess the greatest number of points of excellence, and I hoped to show that porcelain did possess in largest measure the greatest number of points. As far as using porcelain in bicuspids and molars, I believe it is the experience of all that have done this work extensively , and increased the range of their practices, that they are successful in filling the bicuspids and molars. One of our prominent men in Chicago, who is considered a very conservative man, cautioned the members of a society two years ago against using the porcelain in the distal teeth. We left the meeting together, and when on the street I said: "Why did you make such a statement? You know better; you are using it there all the time." He answered: "To tell the truth, I am using porcelain more and more in the molars and bicuspids and I do not know why I do not use it more than I do," The porcelain must be baked right, so that you have no friable edges, but you should not bake the life out of it. Having an absolute fit at all parts of the inlay so that there is a minimum of cement in setting it, you should restore that tooth to almost the full strength it had before caries had made any inroads upon it. As for the gold inlay, it does not bring the beauty to the tooth that porcelain does, and you do not have the success in preserving the pulp of the tooth that you do with porcelain.

Dr. Burke spoke about the time it takes him to make inlays.I believe as he increases his use of porcelain work he will increase the speed with which he manipulates it.

